# A Novel G Protein-Coupled Receptor of *Schistosoma mansoni* (SmGPR-3) Is Activated by Dopamine and Is Widely Expressed in the Nervous System

**DOI:** 10.1371/journal.pntd.0001523

**Published:** 2012-02-28

**Authors:** Fouad El-Shehabi, Amira Taman, Lorena S. Moali, Nelly El-Sakkary, Paula Ribeiro

**Affiliations:** Institute of Parasitology, McGill University, Sainte Anne de Bellevue, Quebec, Canada; Yale School of Public Health, United States of America

## Abstract

Schistosomes have a well developed nervous system that coordinates virtually every activity of the parasite and therefore is considered to be a promising target for chemotherapeutic intervention. Neurotransmitter receptors, in particular those involved in neuromuscular control, are proven drug targets in other helminths but very few of these receptors have been identified in schistosomes and little is known about their roles in the biology of the worm. Here we describe a novel *Schistosoma mansoni* G protein-coupled receptor (named SmGPR-3) that was cloned, expressed heterologously and shown to be activated by dopamine, a well established neurotransmitter of the schistosome nervous system. SmGPR-3 belongs to a new clade of “orphan” amine-like receptors that exist in schistosomes but not the mammalian host. Further analysis of the recombinant protein showed that SmGPR-3 can also be activated by other catecholamines, including the dopamine metabolite, epinine, and it has an unusual antagonist profile when compared to mammalian receptors. Confocal immunofluorescence experiments using a specific peptide antibody showed that SmGPR-3 is abundantly expressed in the nervous system of schistosomes, particularly in the main nerve cords and the peripheral innervation of the body wall muscles. In addition, we show that dopamine, epinine and other dopaminergic agents have strong effects on the motility of larval schistosomes in culture. Together, the results suggest that SmGPR-3 is an important neuronal receptor and is probably involved in the control of motor activity in schistosomes. We have conducted a first analysis of the structure of SmGPR-3 by means of homology modeling and virtual ligand-docking simulations. This investigation has identified potentially important differences between SmGPR-3 and host dopamine receptors that could be exploited to develop new, parasite-selective anti-schistosomal drugs.

## Author Summary

Bloodflukes of the genus *Schistosoma* are the causative agents of human schistosomiasis, a debilitating disease that afflicts over 200 million people worldwide. There is no vaccine for schistosomiasis and treatment relies heavily on a single drug, praziquantel. Recent reports of praziquantel resistance raise concerns about future control of the disease and show the importance of developing new anti-schistosomal drugs. The focus of this research is on the nervous system of the model fluke, *Schistosoma mansoni*. Many pesticides and antiparasitic drugs act by interacting with neuronal proteins and therefore the nervous system is a particularly attractive target for chemotherapeutic intervention. Here we describe a novel receptor of *S. mansoni* that is activated by dopamine, an important neurotransmitter of the schistosome nervous system. The study provides a first in-depth analysis of this receptor and suggests that it plays an important role in the control of muscle function and movement. We also show that the schistosome receptor is substantially different from dopamine receptors of the mammalian host, both in terms of structure and functional properties. We propose that this novel protein could be used to develop new, schistosome-specific drugs aimed at disrupting parasite motility within the host.

## Introduction

The bloodfluke *Schistosoma mansoni* is one of three species of schistosomes that cause significant disease in humans. Approximately 200 million people are infected and another 600 million are at risk of infection. Over 90% of all human schistosomiasis is due to *S. mansoni*. This species exists in Africa, the Middle East, South America and the Caribbean, in regions where the intermediate snail host, *Biomphalaria glabrata*, is also present. There is no vaccine for schistosomiasis and the arsenal of drugs available for treatment is limited. Praziquantel is the drug of choice but concerns over praziquantel resistance [1]–[3] have renewed interest in the search for alternative drug therapies. The nervous system of helminth parasites is considered to be an excellent target for chemotherapeutic intervention. Most of the anthelmintics currently in use, including the mainstay of nematode control, ivermectin, act by interacting with neurotransmitter receptors and cause disruption of neuronal signalling [4]. Recent drug screens conducted on cultured *S. mansoni* suggest that biogenic amine (BA) neurotransmitters may be particularly suitable for development of anti-schistosomal drugs [5], [6]. Substances that normally disrupt BA neurotransmission, such as dopaminergic and serotonergic drugs were shown to halt larval development [5] and to produce aberrant motor phenotypes in culture [6]. The BA systems of schistosomes have not been widely investigated at the molecular level and not much is known about the receptors or other proteins involved. More information is needed to elucidate the mode of action of these neurotransmitters and to identify potential targets for drug discovery.

BAs constitute a group of structurally related amino acid derivatives that function broadly as neurotransmitters and modulators in a variety of organisms. Included in this group are catecholamines (dopamine, noradrenaline, adrenaline), serotonin (5-hydroxytryptamine: 5-HT), histamine and the invertebrate-specific amines, tyramine and octopamine. In flatworms, including *S. mansoni*, BAs play important roles in the control of muscle contraction and movement, activities that are crucial for survival of the parasite within the host [7]–[9]. The best characterized of these amines is serotonin, which is myoexcitatory in all the flatworm species studied to date. Serotonin is synthesized by the parasite [10], it is widely distributed in the nervous system and there is evidence for the existence of a serotonin transport system in *S. mansoni* [11], [12]. At least two putative serotonin receptors are encoded in the *S. mansoni* genome [13], though neither has yet been characterized at the protein level. Besides serotonin, flatworms have both dopamine and histamine within their nervous system [14]–[20]. Dopamine, in particular, has important neuromuscular activities, which can be either excitatory or inhibitory depending on the flatworm species. In *S. mansoni*, dopamine causes relaxation of the body wall muscles [21], possibly by activating a receptor that is associated with neuromuscular structures [22]. In addition to motor effects, BAs have been implicated in the regulation of metabolic activity in several flatworms [8] and recent evidence has shown that serotonin and dopamine are both involved in the transformation of *S. mansoni* miracidia to sporocyst stage [5], suggesting a probable role in parasite development.

BAs exert their effects by interacting with cell-surface receptors, the majority of which belong to the superfamily of G protein-coupled receptors (GPCR) and are structurally related to rhodopsin. GPCRs have a distinctive topology consisting of seven transmembrane (TM) domains separated by loops, the longest of which is the third intracellular loop (il3). Rhodopsin-like (or Class A) GPCRs are further identified by having a relatively short extracelullar N-terminus, which is typically glycosylated, and an intracellular C-terminal tail of variable length [23]. In mammals, BA receptors are classified according to their amine specificity, sequence homology, signalling mechanisms and pharmacological profiles. Each BA interacts with multiple receptors. Dopamine, in particular, interacts with five different receptors (D1–D5), which are classified according to two major structural types, D1- and D2-like [24]. The current annotation of the *S. mansoni* genome has a total of 16 predicted BA receptors, all Class A GPCRs [13]. A few of these sequences, for example the D2-like dopamine receptor of *S. mansoni* (SmD2) [22] share sufficient homology with mammalian prototypes to be classified accordingly. The majority, however, are novel sequences that share about the same level of homology with all different types of BA receptors and can only be defined as BA-like. Among these sequences is a new clade of BA-like GPCRs (named SmGPR) that were previously described in our laboratory [15] and thus far have been detected only in schistosomes. These receptors could prove to be particularly good candidates for selective drug targeting and deserve further investigation.

Two SmGPRs of *S. mansoni* were shown earlier to be functional histamine receptors [15], [25]–[27]. Here we describe a third structurally related receptor (SmGPR-3), which has different agonist specificity. The results presented here show that SmGPR-3 is activated by dopamine and other catecholamines but does not resemble any one particular type of host dopamine receptor, either in terms of overall sequence homology, pharmacological profile or the predicted organization of the binding pocket. Further analysis of the receptor's tissue distribution revealed exceptionally abundant expression throughout the nervous system and suggests an important role for this novel dopamine receptor in neuronal and neuromuscular signalling.

## Materials and Methods

### Parasites

A Puerto Rican strain of *S. mansoni* -infected *Biomphalaria glabrata* snails were kindly provided by Dr. Fred Lewis, Biomedical Research Institute, Rockville, Maryland, USA. *S. mansoni* cercaria were collected from infected snails 35–45 days post-infection. Schistosomula were produced from cercaria by mechanical transformation, as described [27], [28] and were cultured at 37°C and 5% CO_2_ in OPTI-MEM I medium (Invitrogen) supplemented with 10% FBS, streptomycin 100 µg/ml, penicillin 100 U/ml and fungizone 0.25 µg/ml. Adult parasites were obtained by infecting 28-day old female CD-1 mice with freshly collected cercaria (150 cercaria/animal) by skin penetration. Adult *S. mansoni* worms were recovered 7 weeks post-infection by perfusion of the liver [28], washed extensively and either flash-frozen in liquid nitrogen for subsequent RNA extraction or fixed in 4% paraformaldehyde (PFA) for immunolocalization experiments. Animal procedures were reviewed and approved by the Facility Animal Care Committee of McGill University (Protocol No. 3346) and were conducted in accordance with the guidelines of the Canadian Council on Animal Care.

### Cloning of SmGPR-3

The full-length SmGPR-3 cDNA was cloned from adult *S. mansoni* based on a predicted coding sequence (Smp_043290) obtained from the *S. mansoni* genome database (*S. mansoni* GeneDB; http://www.genedb.org/Homepage/Smansoni). Total RNA was purified from adult *S. mansoni* worms (Qiagen RNeasy kit) and was oligo-dT reverse-transcribed with MMLV reverse transcriptase (Invitrogen), according to standard procedures. SmGPR-3 was cloned with primers that targeted the beginning and end of the predicted coding sequence. The primer sequences were as follows: 5′-ATGAATTTCATAAGAAACAAAACCAATTATTC-3′ (sense) and 5′-CTATCTACATCCTTTCAAAAGTACAATATG-3′ (antisense). A proofreading Platinum *Pfx* DNA polymerase (Invitrogen) was used to amplify the cDNA in a standard PCR reaction (35 cycles of 94°C/15 s, 53.1°C/30 s and 68°C/90 s). The resulting amplicon (1,494 bp) was ligated to pGEM-T Easy vector (Promega) and verified by DNA sequencing of two independent clones.

### Yeast functional expression assays

The SmGPR-3 coding sequence was sub-cloned between the *Nco*I/*Xba*I restriction sites of the yeast expression vector Cp4258 (kindly provided by Dr J. Broach, Princeton University, NJ, USA). The functional expression assay was adapted from the protocol of Wang and colleagues [29] as described [15], [30]. The receptor was expressed in *Saccharomyces cerevisiae* strain YEX108 (*MATα* P*_FUS1_-HIS3* P*_GPA1_-Gαq(41)-GPA1-Gaq(5) can1 far1Δ 1442 his3 leu2 lys2 sst2Δ2 ste14::trp1::LYS2 ste18Δ6-3841 ste3Δ1156 tbt1-1 trp1 ura3*; kindly provided by J. Broach, Princeton University, NJ, USA). This strain expresses the *HIS3* reporter gene under the control of the *FUS1* promoter and contains an integrated copy of a chimeric Gα gene in which the first 31 and last five codons of native yeast Gα (GPA1) were replaced with those of human Gα_q_ [29]. Other strains carrying chimeras of GPA1 and human Gα_i2_, Gα_12_, Gα_o_ or Gα_s_ were tested in preliminary experiments but were found to yield lower or no receptor activity compared with strain YEX108. YPD culture medium (1% yeast extract, 2% peptone and 2% dextrose) was used to culture YEX108 strain, according to standard conditions. The lithium acetate method was performed to transform yeast with either empty Cp4258 vector (mock) or Cp4258/SmGPR-3 expression plasmid, using 200 µl mid-log phase cells, 200 µg carrier single stranded DNA (Invitrogen) and 1 µg plasmid. Positive transformants were selected on synthetic complete (SC) 2% glucose solid medium lacking leucine (SC/leu^−^). For agonist assays, single colonies of transformants were cultured overnight in SC/leu^−^ liquid medium at 250 rpm/30°C. The next day, cells were washed four times in SC 2% glucose liquid medium that lacked both leucine and histidine (SC/leu^−^/his^−^). Cells were finally resuspended in SC/leu^−^/his^−^ medium supplemented with 50 mM 3-(N-morpholino) propanesulfonic acid (MOPS), pH 6.8 and 1.5 mM 3-Amino-1, 2, 4-Triazole (3-AT). The latter was used to reduce basal growth due to endogenous background signalling as it inhibits the gene product of *HIS3* [29]. Aliquots containing approximately 3,000 cells were added to individual wells of a 96-well plate containing test agonist or vehicle plus additional medium for a total reaction volume of 100 µl. The plates were incubated at 30°C for 22–26 h, after which 10 µl of Alamar blue (Invitrogen) was added to each well. The plates were returned to the 30°C incubator until the Alamar blue color started to shift to pink (approximately 1–4 h) and fluorescence (560 nm excitation/590 nm emission) was measured at 30°C every hour up to three hours, using a plate fluorometer (FlexStation II, Molecular Devices, US). Antagonist assays were done in a similar way, except that each well contained 10^−4^ M agonist (dopamine) and the antagonist at the specified concentration. Data analyses and dose-response curve fits were performed using Prism v5.0 (GraphPad software Inc.).

### SmGPR-3 antibody production

Polyclonal antibodies were produced in rabbits against two SmGPR-3 synthetic peptides. The first peptide (CYISYSKEYRIYSSV) is located in the predicted 2^nd^ extracellular loop (ECL2) of SmGPR-3 (positions 186–200) and the second peptide sequence (CERKTERTIKTQRQF) is in the third intracellular loop (il3) (positions 395–409). The two peptide sequences were tested against the *S. mansoni* GeneDB database and the general database at The National Center for Biotechnology Information (NCBI) to insure specificity. The peptides were both conjugated to ovalbumin to increase immunogenicity. The conjugated peptides and custom antibodies were purchased from 21^st^ Century Biochemicals, Malboro, MA, USA. Antibodies were raised in two rabbits, each of which was inoculated five times and the serum was collected prior to injection (preimmune) and up to 72 days following injection (see http://21stcenturybio.com/ for further details of the antibody protocol). ELISA was done to test the specificity of the antibodies to each of the two peptides. SmGPR-3 –specific antibodies were subsequently affinity-purified, using the MicroLink Peptide Coupling kit (Pierce), as described previously [22].

### Immunoprecipitation and western blotting

Immunoprecipitation (IP) was performed with the Seize Primary Immunoprecipitation kit (Pierce, USA), as described previously [22]. Briefly, IP affinity columns were prepared by covalent coupling of purified anti-SmGPR-3 IgG to AminoLink Plus gel in the presence of sodium cyanoborohydride. A solubilized membrane fraction was prepared from adult *S. mansoni*, using a commercial kit (ProteoExtract Native Membrane Protein Extraction Kit, Calbiochem) and aliquots of solubilized membrane proteins (14 µg protein) were mixed with 50 µl IgG-linked gel and incubated overnight at 4°C with gentle rotation. After incubation, the gel was washed extensively and the bound proteins were eluted under acidic conditions, using the elution buffer supplied by the kit. The immunoprecipitated proteins were resolved on 4–12% Tris-Glycine precast gel (Invitrogen) and transferred to polyvinylidene fluoride (PVDF) membranes (Millipore). Western blot analysis was performed according to standard protocols, using purified anti-SmGPR-3 antibody (dilution 1/10,000) and a goat anti-rabbit Horseradish Peroxidase (HRP)-conjugated antibody (Calbiochem) (1/20, 000). To test for specificity, the western blots were repeated with primary antibody that was preadsorbed with 0.5 mg/ml of pooled peptide antigens (0.25 mg of each peptide) or preimmune serum.

### Confocal immunofluorescence

The procedure is based on the protocol of D. Halton and colleagues [31], as described previously [22], [27]. *S. mansoni* cercaria and adult worms were fixed in 4% paraformaldehyde (PFA) for 4 h at 4°C, washed three times in blocking buffer (1× PBS, pH 7.4; 1% bovine serum albumin; 0.1% sodium azide and 0.5% triton X-100) and were incubated in 10 mM sodium citrate for 1 h at 70°C. Animals were subsequently washed twice with PBS, incubated with affinity-purified anti-SmGPR-3 antibody (diluted 1∶25 in blocking buffer) for three days at 4°C, washed overnight with the same blocking buffer and finally incubated with goat anti-rabbit IgG conjugated to FITC (Sigma, Canada) for another three days at 4°C (dilution 1∶300). If used as a counterstain, 200 µg/ml TRITC-labelled phalloidin was added during the last two days of incubation. The samples were mounted using anti-quench mounting medium (Sigma, Canada) and examined with a BIO-RAD RADIANCE 2100 confocal laser scanning microscope (CLSM) equipped with a Nikon E800 fluorescence microscope for confocal image acquisition and the LASERSHARP 2000 software package. As negative controls, we used preimmune serum, omitted the primary antibody and used primary antibody that was preadsorbed with 0.5 mg/ml of pooled peptide antigens (0.25 mg of each peptide).

### Measurements of motor activity

3-day old *in vitro* transformed schistosomula were placed in individual wells of a 24-well plate (30–40 animals/well) in 300 µl of OPTI-MEM+10% dialyzed serum. Following an adaptation period of 15 min at room temperature, test substances were added at a final concentration of 100 µM or as indicated. Animals were monitored 5 min after drug addition by placing the 24-well plate on a compound microscope (Nikon, SMZ1500) equipped with a digital video camera (QICAM Fast 1394, mono 12 bit, Qimaging) and SimplePCI version 5.2 (Compix Inc.) for image acquisition. Images were obtained at a rate of ≈3 frames/s for a period of 1 minute and the data were analyzed with ImageJ software (version 1.41, NIH, USA). Cultured schistosomula display complex motor behaviours that are dominated by repeated changes in length, both shortening and elongation. To quantify this type of movement, we used the “Fit-Ellipse” command of ImageJ to draw best-fit ellipses of individual animals in each recorded frame. An estimate of body length was obtained by measuring the principal (“major”) axis of each ellipse, using calibrated units (µm), and the frequency of length changes during the observation period was calculated. Any change representing >10% of body length, whether an increase or decrease, was included in the calculation; changes ≤10% were disregarded. Between 12–15 animals were monitored per well and the experiment was repeated a minimum of 3 times. To monitor for possible drug induced toxicity, viability tests were performed routinely using the methylene blue dye exclusion assay described by Gold [32].

### Bioinformatics analyses

Homology searches were performed by BLAST analyses (tBLASTn or BLASTp) of the *S. mansoni* Genome Database (*S. mansoni* GeneDB; www.genedb.org/genedb/smansoni/) [13], the *S. japonicum* Transcriptome and Proteome Database (SjTPdb) [33], the most current genome annotations of the planarians, *Schmidtea mediterranea* (SmedGD version 1.3.14) [34] and *Macrostomum lignano* (www.macgenome.org/index.html) and the general database available at the National Center for Biotechnology Information (NCBI). Sequences showing significant homology with SmGPR-3 were aligned with ClustalW and inspected manually for the presence of conserved Class A (rhodopsin-like) GPCR motifs [23]. Phylogenetic trees were generated with MEGA4 [35], using two different methods, neighbour-joining and Unweighted Pair Group Method with Arithmetic mean (UPGMA) with similar results. The trees were tested by bootstrap analysis with 1,000 replicates. Predictions of transmembrane (TM) regions were made using the TMpred server (http://www.ch.embnet.org) and by comparison with the crystal structure of the human β2 adrenergic GPCR [36]. To facilitate identification, *S. mansoni* sequences are described using both their *S. mansoni* GeneDB designation [13] and the corresponding GenBank accession numbers. *S. mediterranea* sequences are identified by their SmedGD designation [34]. All other sequences are identified by their GenBank accession numbers. GPCR residues located within TM regions are described according to the system of Ballesteros and Weinstein [37]. Each amino acid within a TM region is identified by the TM number (1–7) followed by the position in the TM helix relative to an invariant reference residue, which is arbitrarily assigned the number 50. Residue D^3.32^, for example, is located in TM3, 18 residues upstream of the invariant reference residue. Amino acids of relevance to this study are as follows (corresponding residue of SmGPR-3): R^2.64^ (Arg96), D^3.32^ (Asp117), S^5.42^ (Ser198), S^5.43^ (Ser199), T^7.39^ (Thr462) and Y^7.43^ (Tyr466).

### SmGPR-3 modeling and ligand docking

A theoretical model of SmGPR-3 was built with Accelrys Discovery Studio (DS). Prior to generating the model, SmGPR-3 was aligned with the sequences of GPCR crystal structures available in the general protein database (PDB) (Accession numbers: 2rh1, 3eml, 1u19, 2vt4, 2z73) and the β-2 adrenergic receptor (2hr1) was selected as the best template based on similarity scores. The sequence alignment was inspected to ensure that the positions of conserved residues in the structural template were properly aligned with those of SmGPR-3, including the reference residues designated at position 50 of each helix [37] and conserved motifs, such as the DRY peptide at the end of TM3 and the NPxxY motif of TM7. Subsequent preparation of the template and construction of the model were performed with DS using default parameters. The first stretch of 28 amino acid residues corresponding to the extracellular N-terminal end was not constructed due to lack of structural information. In addition, we note that the β-2 adrenergic template is a chimeric receptor in which the region corresponding to the third intracellular loop (il3) was replaced with the sequence of T4 lysozyme [36]. Therefore the il3 of SmGPR-3 (positions 224–417) could not be aligned and was omitted from the model. Energy minimization was performed using the CHARMm forcefield in DS. The conserved disulfide linkage that occurs between the beginning of TM3 and extracellular loop 2 (Cys110 and Cys186 in SmGPR-3) was constrained during optimization of the model. The energy-minimized model was subsequently verified by means of a Ramachandran plot analysis and the PROFILES-3D evaluation method available in DS. The quality score obtained with PROFILES-3D was within acceptable range and the Ramachandran plot analysis showed 94.8% of the residues occurring in favourable regions of the plot, suggesting the model was reliable. Superimposition of the model with the β-2 adrenergic receptor template showed a backbone (C_α_) root-mean-square-deviation (RMSD) of 0.83 Å and the overall protein RMSD was 1.38 Å. For ligand-docking, the structure of the ligand (dopamine or epinine) was generated with the molecular builder panel available in the software and was energy-minimized, as recommended. Next, we used DS to search for potential binding cavities. Six potential sites were identified, of which only one was located in the correct region based on the position of the binding pocket in the structural template. The ligand was subsequently docked onto this site of the SmGPR-3 model with CDOCKER. Each ligand was docked in multiple conformational states and orientations, which resulted in 240 different ligand poses for dopamine and 290 for epinine. These were examined and evaluated using the CHARMm scoring method of CDOCKER to identify potential binding residues for each ligand.

### Other methods

Protein content was measured with a Lowry assay (BioRad). Indirect ELISA was performed in 96-well plates coated with individual or pooled SmGPR-3 peptides (50–500 ng/well) and incubated with a serial dilution of rabbit anti-SmGPR-3 antiserum or preimmune serum (1∶30,000–1∶100), followed by incubation with a horseradish peroxidase (HRP)-labeled secondary antibody (goat anti-rabbit IgG, 1∶2,000). Quantitative PCR (qPCR) was performed as described previously [15], [52] using the Platinum SYBR Green qPCR SuperMix-UDG kit (Invitrogen) and a Rotor-Gene RG3000 (Corbett Research) real-time PCR cycler. Statistical comparisons were done with Student *t*-tests or a one-way ANOVA, followed by a Tukey pairwise comparison. *P*≤0.05 was considered statistically significant.

## Results

### SmGPR-3 belongs to a new clade of schistosome BA-like receptors

**Figure 1:**
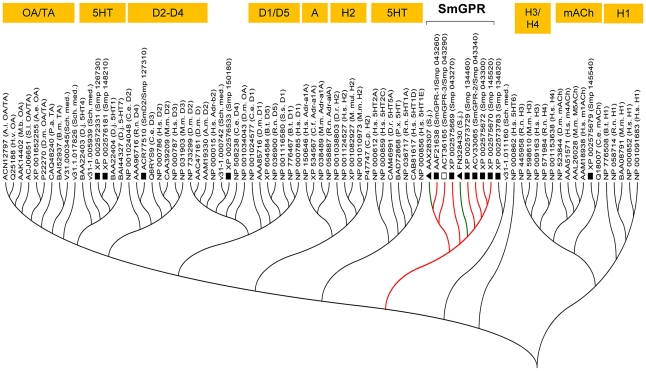
Dendogram analysis of biogenic amine (BA) G protein-coupled receptors (GPCR). A rooted phylogenetic tree was constructed from a ClustalW sequence alignment of vertebrate and invertebrate BA receptors, using MEGA 4 [35]. Included in the alignment are 15 predicted *Schistosoma mansoni* and *S. japonicum* BA GPCR sequences, of which nine clustered together into a separate clade (SmGPR). The receptor described in this paper, SmGPR-3 is identified by an open square (□). Other *S. mansoni* receptors are marked with solid squares (▪) and *S. japonicum* receptors are marked with solid triangles (▴). Sequences are identified by their accession numbers and the species names are abbreviated as follows: A.e. (*Aedes aegypti*), A.i. (*Agrotis ipsilon*), A.m. (*Apis mellifera*), B.m. (*Bombyx mori*), B.t. (*Bos taurus*), C.e. (*Caenorhabditis elegans*), C.f. (*Canis familiaris*), C.p. (*Cavia porcellus*), D.m. (*Drosophila melanogaster*), D.j. (*Dugesia japonica*), D.r. (*Danio rerio*), H.s. (*Homo sapiens*), H.v. (*Heliothis virescens*), M.b. (*Mamestra brassicae*), M.m. (*Mus musculus*), M.mul. (*Macaca mulatta*), P.a. (*Periplaneta americana*), P.x. (*Papilio xuthus*), R.n. (*Rattus norvegicus*), S.j. (*S. japonicum*), S.med. (*Schmidtea mediterranea*), S.l. (*Spodoptera littoralis*) and S.s. (*Sus scrofa*). Predicted *S. mansoni* coding sequences are identified by their “Smp” designation obtained from the *S. mansoni* Genome database (*S. mansoni* GeneDB) and the corresponding GenBank Accession number. H1–H4, histamine type 1–4 receptors; D1–D5, dopamine type 1–5 receptors; A, adrenergic receptors; 5HT, serotonin (5-hydroxytryptamine) receptors; mACh, muscarinic acetylcholine receptors; OA/TA, octopamine/tyramine receptors.

Predicted *S. mansoni* BA-like receptors [13] were aligned with BA receptors from other species, including other flatworms for which genomic data are available (*S. japonicum*, *Dugesia japonica* and *S. mediterranea*) and both vertebrate and invertebrate representatives of dopaminergic, serotonergic, adrenergic, histaminergic, tyramine/octopamine and structurally related cholinergic muscarinic (mACh) receptors. A phylogenetic tree of the alignment (Fig. 1) shows a subset of schistosome GPCRs (SmGPR) that are derived from a common node and constitute a separate clade within the tree. Included in this clade are seven *S. mansoni* sequences and two homologues from *S. japonicum* but no sequences from any of the other species examined, including the free-living planarians. We have previously described two members of this new clade, SmGPR-1 (formerly SmGPCR; AAF21638; Smp_043260;) and SmGPR-2 (GQ397114; Smp_043340) [15], [25]–[27]. In the present study, we cloned a third SmGPR (SmGPR-3) cDNA from adult *S. mansoni* by RT-PCR. The cDNA was verified by DNA sequencing (Accession # GQ259333) and was found to be identical to the corresponding genomic prediction available at the *S. mansoni* GeneDB (Smp_043290). SmGPR-3 has 497 amino acids and a predicted MW of 58.4 kDa.

**Figure 2:**
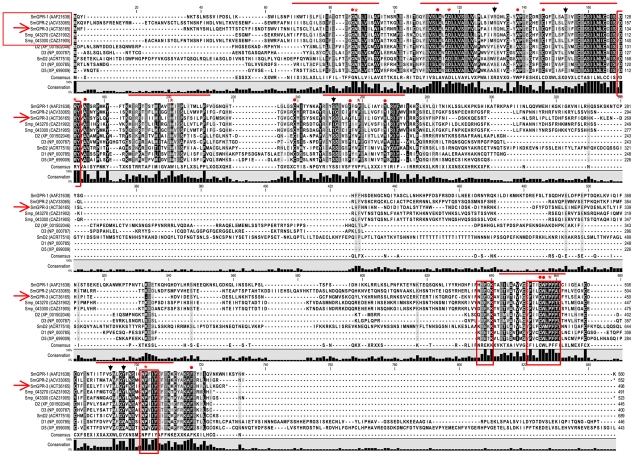
Sequence alignment of dopaminergic G protein-coupled receptors with *Schistosoma mansoni* SmGPR receptors. A ClustalW alignment was performed using representative examples of vertebrate dopaminergic GPCRs (D1–D5), the *S. mansoni* dopamine D2-like receptor (SmD2) and several members of the SmGPR clade. SmGPR sequences are boxed (horizontal box) and SmGPR-3 is marked by an arrow. Receptor sequences are identified by their accession numbers (brackets). The positions of the predicted seven transmembrane domains are marked by horizontal lines and the invariant residue in each TM segment [37] is identified by an asterisk (*) Other conserved residues of functional relevance are marked by circles (•) and conserved motifs are boxed (vertical boxes). Residues discussed in this study, R^2.64^ (Arg96), D^3.32^ (Asp117), S^5.42^ (Ser198), T^7.39^ (Thr462) and Y^7.43^ (Tyr466) are identified by vertical arrows.

NCBI BLASTp analyses confirmed the identity of SmGPR-3 as a member of the BA receptor family. According to pairwise alignment analyses, the most closely related sequences are those of the SmGPR clade including (% homology): SmGPR-1 (Smp_043260, 53.4%), Smp_043300 (47.4%), SmGPR-2 (Smp_043340, 46.9%), Smp_043270 (45.5%), Smp145520 (40.4%) and the *S. japonicum* receptor FN328430 (46.1%). SmGPR-3 is also related to other schistosome BA receptors (non-SmGPRs), as well as BA receptors from other organisms but the level of homology is generally lower (<40%). Further analysis of the SmGPR-3 protein sequence detected all the hallmark features of Class A (rhodopsin-like) GPCRs (Fig. 2). Aside from having the expected 7-TM topology, SmGPR-3 carries the signature DRY motif at the intracellular boundary of TM3, the NPxxY motif of TM7 and all the conserved reference residues at position #50 of each TM helix [37]. We also identified several residues that have been implicated in BA binding and receptor activation, notably the aromatic cluster FxxCWxPFF of TM6 and a highly conserved aspartate at position 3.32 of TM3 (D^3.32^/Asp117), which is considered to be one of the core binding sites in BA GPCRs [23], [36], [38], [39]. The presence of D^3.32^ marks an important difference between SmGPR-3 and other members of this clade. The schistosome SmGPRs are unusual in that they carry an asparagine substitution at this position [15]. SmGPR-3 is the only receptor in this group where the aspartate D^3.32^ is conserved.

### SmGPR-3 is a dopamine/catecholamine receptor

**Figure 3:**
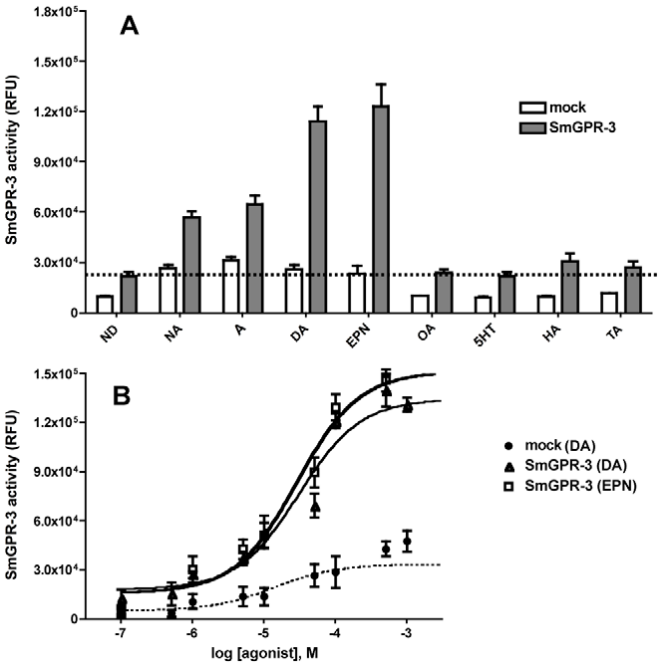
Functional expression of the *Schistosoma mansoni* SmGPR-3 receptor in yeast. (A) The full-length SmGPR-3 cDNA was expressed in *Saccharomyces cerevisae* strain YEX108 and grown in selective leu/histidine-deficient (leu^−^/his^−^) medium containing 2×10^−4^ M of each biogenic amine or vehicle (no drug control, ND). Yeast cells transformed with empty plasmid were used as a negative control (mock). Receptor activation was quantified from measurements of yeast growth in relative fluorescence units (RFU), using an Alamar blue fluorescence assay. The results are the means ± S.E.M. of 5–6 independent clones, each assayed in triplicate. The following biogenic amines were tested: adrenaline (A), noradrenaline (NA), dopamine (DA), epinine (EPN), serotonin (5-hydroxytryptamine, 5HT), octopamine (OA), tyramine (TA) and histamine (HA). (B) Functional assays were repeated with the same SmGPR-3-expressing yeast strain and variable concentrations of DA (△) or EPN (□). The mock control was tested with DA (•). EC_50_ values for DA and EPN are 3.10×10^−5^ M and 2.85×10^−5^ M, respectively. The data are the means ± S.E.M. of two experiments, each in triplicate.

Functional expression assays were performed in yeast. Preliminary experiments failed to detect receptor expression in mammalian cells (data not shown) and therefore we used yeast as a heterologous expression system throughout the study. The full-length SmGPR-3 cDNA was ligated to the Cp4258 vector and the recombinant plasmid was transformed into *Saccharomyces cerevisae* to test for receptor activity. We used a genetically modified *S. cerevisae* strain that is designed for GPCR activity assays [29]. The yeast is auxotrophic for histidine and expresses a *HIS3* reporter gene under the control of the *FUS1* promoter. Activation of a recombinant GPCR in this system increases expression of the *HIS3* reporter via the yeast's endogenous pheromone response, which in turn allows the cells to grow in selective histidine-deficient medium. Thus receptor activity can be quantified based on measurements of yeast growth in the selective medium, using a fluorometric Alamar Blue assay. Cells transformed with either empty vector (mock control) or SmGPR-3 were initially tested with all different biogenic amines, each at 2×10^−4^ M (Fig. 3A). The results obtained from five to six individual clones showed that SmGPR-3 was selectively activated by dopamine and its naturally occurring metabolite epinine (deoxyepinephrine). Other catecholamines, including noradrenaline, adrenaline and the adrenaline metabolite, metanephrine (not shown) also stimulated SmGPR-3 activity but not to the same extent as dopamine or epinine. The receptor exhibited partial constitutive activity in the absence of agonist but there was further activation in the presence of catecholamines, whereas other biogenic amines had no significant effect relative to the no drug control. Experiments were repeated with different concentrations of dopamine or epinine and their responses were shown to be dose-dependent. The half maximal effective concentration (EC_50_) for dopamine and epinine activation in the yeast expression system are 3.1×10^−5^ M and 2.85×10^−5^ M, respectively (Fig. 3B).

### SmGPR-3 has atypical pharmacology

**Figure 4:**
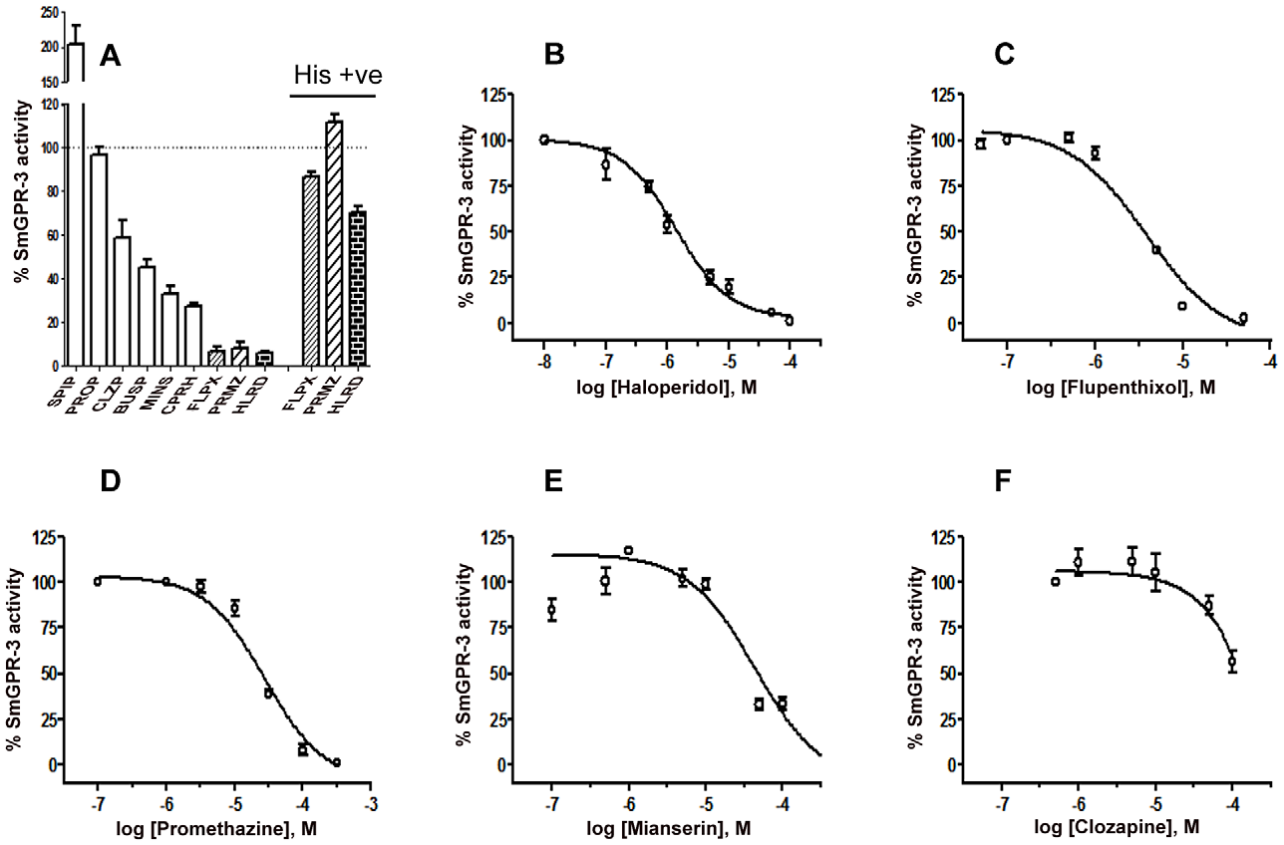
Antagonist effects on SmGPR-3 activity. (A) Yeast YEX108 auxotrophic *his* strain expressing SmGPR-3 was incubated with agonist (DA, 100 µM) and test antagonist or vehicle. Antagonists were tested at 100 µM except for flupenthixol, which was used at 10 µM. The data were normalized relative to the control sample that contained 100 µM DA but no antagonist. To test for drug induced toxicity, assays were repeated in the presence of 100 µM test antagonist in histidine-supplemented (*his+*) medium, which enables the cell to grow irrespective of receptor activation (His +ve control; see text for details). Abbreviations are as follows: SPIP, spiperone; PROP, propanolol; CLZP, clozapine; BUSP, buspirone; MINS, mianserin; CPRH, cyproheptadine; FLPX, flupenthixol; PRMZ, promethazine; HLRD, haloperidol. B–F. Dose-dependent inhibiton by haloperidol (IC_50_ = 1.4 µM), flupenthixol (IC_50_ = 3.9 µM), promethazine (IC_50_ = 28.0 µM), mianserin (IC_50_ = 45.0 µM) clozapine (IC_50_>100 µM). The error bars are the means ± SEM for 3–4 experiments and at least 2 clones (in triplicates).

Next we examined the effects of classical (mammalian) dopaminergic and other BA antagonists on the activity of SmGPR-3. Drugs were tested initially at a single concentration of 100 µM (10 µM in the case of flupenthixol) in the presence of 100 µM DA (Fig. 4A). The drug effects revealed an unusual pharmacological profile, which did not resemble any of the dopaminergic or adrenergic receptors of mammals. The most surprising observation was that spiperone, a mammalian D_2_ antagonist enhanced the activity of SmGPR-3 nearly 2-fold, thus behaving more as an agonist than a receptor blocker. Propranolol, a β-adrenoceptor antagonist had no effect on this receptor, while the remaining drugs showed various degrees of inhibition. Those drugs that produced significant inhibition (>50%) in the initial screen were subsequently tested at different concentrations to obtain dose response curves (Fig. 4B–F). The half-maximal inhibitory concentrations (IC_50_) for these drugs were as follows: Haloperidol, 1.4×10^−6^ M; Flupenthixol, 3.9×10^−6^ M; Promethazine, 2.8×10^−5^ M; Mianserin, 4.5×10^−5^ M; Clozapine >10^−4^ M. Based on this analysis, the most effective antagonists of SmGPR-3 were haloperidol and flupenthixol, two classical DA antagonists, followed by promethazine, an antihistaminic drug not known to interact with dopaminergic receptors. Mianserin, (mixed adrenergic/5HT antagonist) and cyproheptadine, (mixed histamine/5-HT antagonist) both produced significant inhibition of ≈70% at the highest concentration tested. Finally the remaining drugs caused modest or no significant inhibition at 100 µM, including clozapine, a classical dopamine antagonist. Because the assay is based on cell growth, we questioned whether the strong inhibition induced by promethazine, flupenthixol and haloperidol were due to drug-induced toxicity leading to cell death. To test this possibility, we repeated the assay in medium supplemented with histidine (100 µM), which enables cell growth irrespective of receptor activation. The results showed normal or nearly normal growth in the drug-treated cells in the presence of histidine, indicating that the inhibitory effect of the drug was receptor-mediated and not the product of generalized toxicity (Fig. 4A).

### Immunolocalization of SmGPR-3

**Figure 5:**
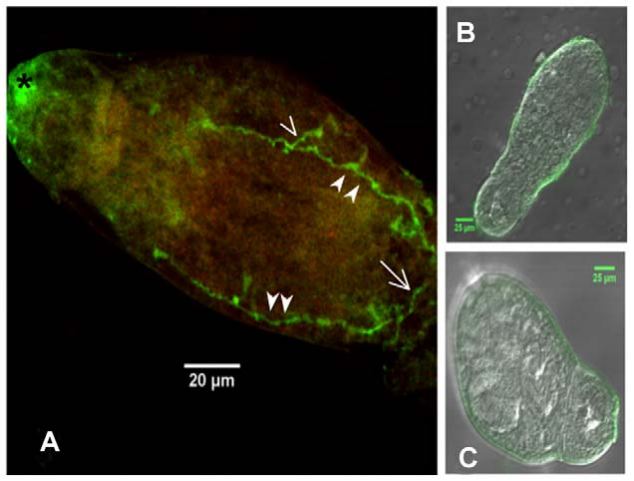
Immunolocalization of SmGPR-3 in larval *Schistosoma mansoni*. *S. mansoni* cercaria were probed with affinity purified anti-SmGPR-3 antibody, followed by fluorescein isothiocyanate (FITC)-labelled secondary antibody. (A) Immunoreactivity (green) can be seen along the major longitudinal nerve cords (solid arrowheads) and in transverse commissures (open arrowhead), including the posterior transverse commissure near the base of the tail (open arrow). (B) No significant immunoreactivity was observed in negative controls probed with anti-SmGPR-3 antibody that was pre-adsorbed with peptide antigens or (C) controls probed with secondary antibody only. (*) non-specific labelling.

**Figure 6:**
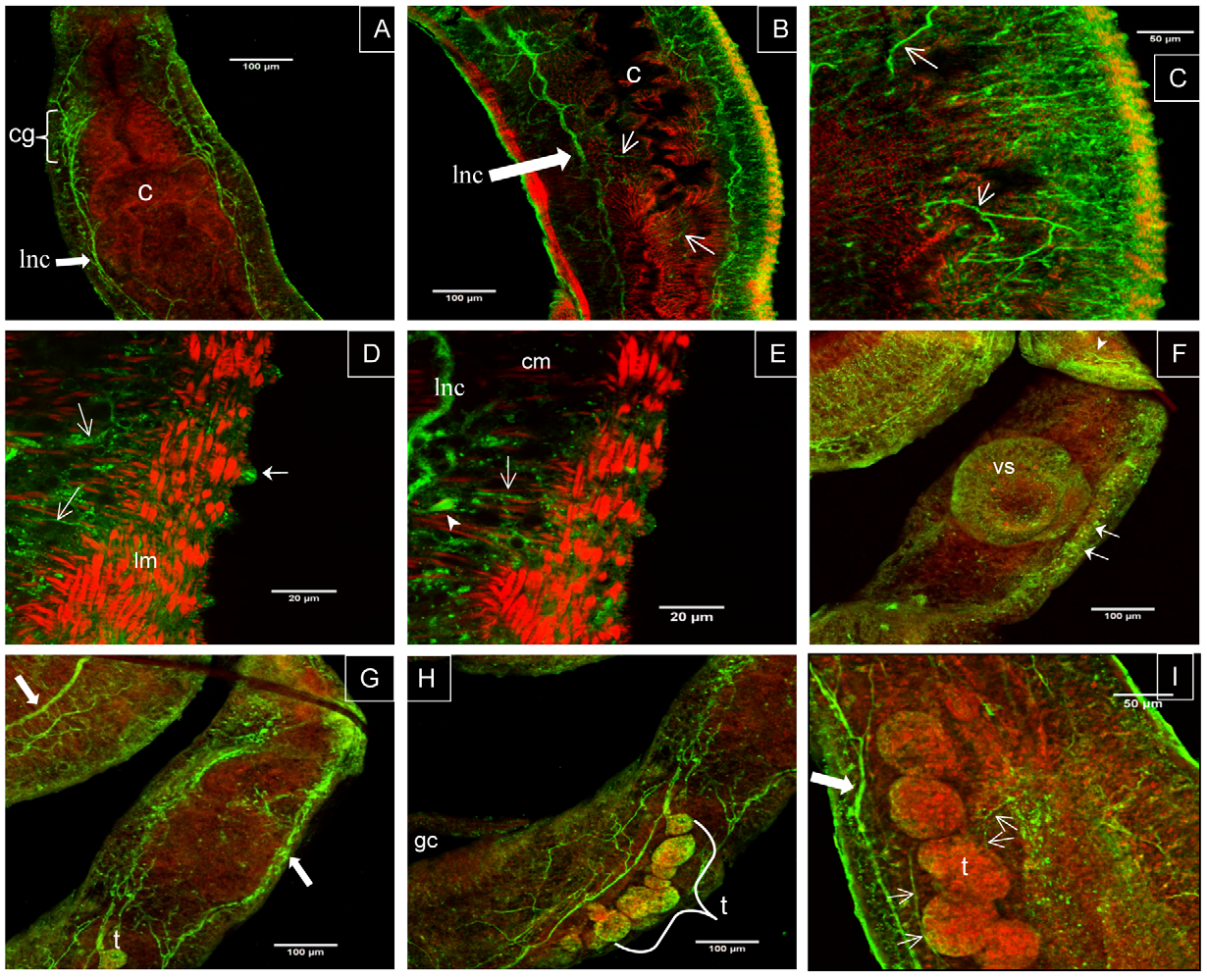
Immunolocalization of SmGPR-3 in adult *Schistosoma mansoni*. Adult male worms were treated with affinity-purified anti-SmGPR-3 polyclonal IgG, followed by fluorescein isothiocyanate (FITC)-labelled secondary antibody (green). Animals were counterstained with tetramethylrhodamine B isothiocyanate (TRITC)-labelled phalloidin (red) to visualize the musculature of the body wall and digestive tract. (A) Strong SmGPR-3 immunoreactivity is visible in the region of the cerebral ganglia (cg) and along the main longitudinal nerve cords (lnc) of the CNS. (B) Green fluorescence can also be seen in peripheral nerve fibers innervating the caecum (open arrows). (C) Near the surface SmGPR-3 is strongly expressed in the peripheral innervation of the body wall muscles and the tegument. Numerous immunoreactive nerve fibers (open arrows), some varicose in appearance, are visible throughout this region. At higher magnification (D, E) we see SmGPR-3–expressing nerve fibers (open arrows) and cell bodies (solid arrowhead) innervating the body wall muscles, both circular muscle (cm) and typically spindle-shaped longitudinal muscle fibers (lm). SmGPR-3 immunoreactivity is seen in the tubercles of male worms (D, solid arrow), where it is probably associated with sensory nerve endings. (F) SmGPR-3 is expressed in the nerve plexus and small fibers of the ventral sucker. Extensive labelling of the submuscular nerve plexus can also be seen in this specimen (solid arrows). (G, H) A male worm showing strong labelling of major nerve cords (solid arrows) and the reproductive tract, including the testes (t) and associated nerves. (I) Fine SmGPR-3 immunoreactive nerve fibers (open arrows) innervate the testicular lobes of male worms. lnc, longitudinal nerve cords; cg, cerebral ganglia; c, caecum; lm, longitudinal muscle; cm, circular muscle; vs, ventral sucker; t, testes; gc, gynecophoral canal.

To investigate the tissue localization of SmGPR-3 we obtained a specific antibody that targets two unique peptides of the receptor. The antibody was affinity-purified and verified first by ELISA. To test if the antibody could recognize the native receptor, we immunoprecipitated (IP) SmGPR-3 from solubilized *S. mansoni* membranes, using covalently-coupled anti-SmGPR-3 antibody beads and then probed the IP eluate by western blotting with affinity-purified anti-SmGPR-3 antibody. The results (Supplemental Fig. S1) detected a single major band of about 60 kDa, which is consistent with the expected size of SmGPR-3. The negative controls were similarly immunoprecipitated with the same antibody beads but were probed either with peptide-preadsorbed antibody or preimmune serum. The results show much diminished or no immunoreactivity in the negative controls, suggesting the antibody recognizes SmGPR-3 specifically. For the *in situ* immunolocalization studies, larval and adult stages of *S. mansoni* were probed with affinity-purified anti-SmGPR-3, followed by a FITC-labelled secondary antibody. Some animals were also treated with TRITC-conjugated phalloidin to label cytoskeletal elements and muscle [31]. The results show strong SmGPR-3 green fluorescence in the nervous system of both cercaria and adult *S. mansoni*. In cercaria we see immunoreactivity primarily in the CNS along the main longitudinal nerve cords and transverse commissures (Fig. 5). The pattern of labelling is similar to that of dopamine itself, which localizes to the same regions of the CNS in cercaria of both *S. mansoni* and *S. japonicum* [14]. Adult worms have high levels of SmGPR-3 immunoreactivity in the cerebral ganglia and major longitudinal nerve cords. This was observed in adult males (Fig. 6A) as well as female worms (not shown). The peripheral nervous system (PNS) is also rich in SmGPR-3. Immunoreactivity can be seen in the innervation of the caecum (Fig. 6B) and the peripheral plexuses and fibers innervating the parasite musculature (Fig. 6C), both circular and longitudinal muscles (Fig. 6D, E). There is no apparent co-localization of SmGPR-3 labelling (green) and the somatic muscles (red) that were counterstained with TRITC-conjugated phalloidin, suggesting the receptor is probably associated with the innervation of the musculature rather than the muscle itself. Other regions of significant labelling include the peripheral plexus of the ventral sucker (Fig. 6F) and, in male worms, the tubercles and the innervation of the testes (Fig. 6G–I). No significant fluorescence was observed in any of the negative controls tested, including worms probed with pre-immune serum, secondary antibody only and antibody that was pre-adsorbed with the peptide antigens.

### *In vitro* motility assays

**Figure 7:**
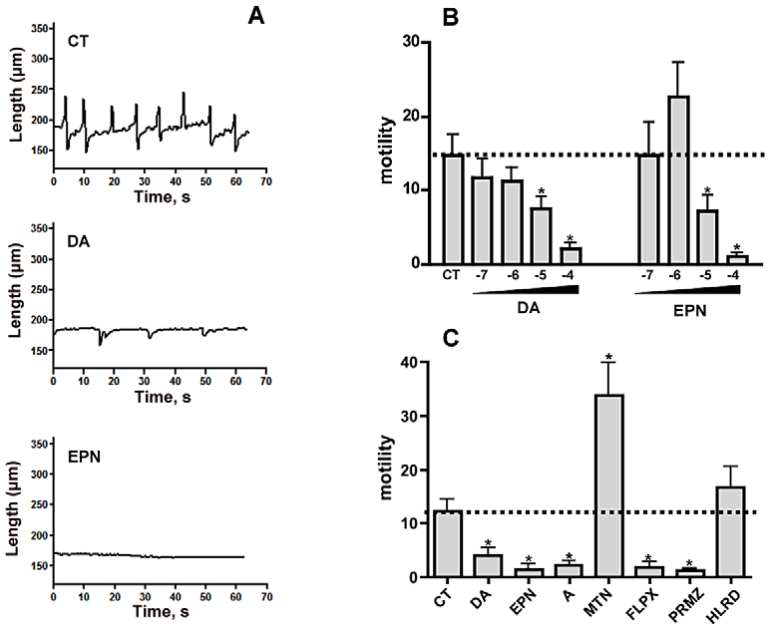
Effects of dopamine and related substances on schistosome motility. (A) *In vitro* transformed 3-day-old schistosomula were incubated with test drug, dopamine (DA) or epinine (EPN), each at (10^−4^ M) or vehicle (CT, control). Animals were treated for 5 min at room temperature, after which they were examined with a compound microscope equipped with a digital video camera and SimplePCI (Compix Inc.) for image acquisition. Images were recorded for 1 minute (∼3 frames/second) and an estimate of body length in µm was obtained for each animal in every frame. Each tracing shown is of an individual animal and is representative of 12–15 larvae per experiment and 3–4 independent experiments per treatment. (B) Experiments were repeated with various concentrations of test agonist in a range of 10^−7^ M–10^−4^ M, or in the absence of test substance (CT, control). Images were recorded as above and body length was measured for each frame. Motility is defined as the frequency of length changes (shortening and elongation) per minute of observation, as described in the Methods. The data are presented as the means and SEM of three separate experiments each with 12–15 animals. (C) Schistosomula were treated with test substances at a single concentration or in the absence of drug (CT, control) and motility was measured as above. Dopamine (DA), epinine (EPN), flupenthixol (FLPX), promethazine (PRMZ) were each tested at 50 µM. The remaining substances, adenaline (A), metanephrine (MTN) and haloperidol (HLRD) were tested at 500 µM. The data are the means and SEM of three separate experiments each with 12–15 animals. * Significantly different from the no drug control at P<0.05.

The prevalence of SmGPR-3 in the peripheral innervation of the somatic muscles suggests this receptor could be involved in the control of motor activity. To explore this possibility we tested the effects of several SmGPR-3 agonists and antagonists on the motor activity of intact schistosomula in culture. The goal of these studies was to determine whether substances that interact with the recombinant receptor *in vitro* also influence worm movement, which could suggest involvement of SmGPR-3 in motor control. We used a larval stage (schistosomula) instead of adult worms because the larvae are better suited for quantitative measurements of movement. A preliminary analysis by confocal immunofluorescence detected expression of SmGPR-3 in the nervous system of the schistosomula (data not shown). We also determined that SmGPR-3 could be amplified from schistosomula oligo-dT reverse-transcribed cDNA using both end-point and real-time quantitative PCR (Supplemental Fig. S2), thus confirming that the receptor is expressed in this larval stage. Measuring schistosome movement is challenging because the worms do not travel (i.e. swim) in culture. To quantitate motor activity, we monitored individual schistosomula in the presence and absence of test substances, using a microscope equipped with a video camera and imaging software. Approximately 180 consecutive frames were recorded for each animal over 1 min of observation and the approximate length of the animal in each frame was measured in calibrated units (µm). An individual recording of a control (untreated) animal is shown in Fig. 7A (top panel). When cultured *in vitro*, schistosomula exhibit repeated cycles of elongation and contraction, which cause the animal to increase or decrease its body length by as much as 20% in either direction. Treatment with catecholamines significantly altered this behaviour. The addition of dopamine or epinine, each at a concentration of 100 µM caused marked inhibition of motor activity. Dopamine decreased both the amplitude and frequency of contractions and epinine caused virtual paralysis in all animals tested. To quantify the level of motor activity, we repeated experiments at different concentrations of each amine and then calculated the frequency of length changes, both decrease and increase for each individual animal during the 1 min of observation. Mean values were obtained from the average of 12–15 animals/experiment and each experiment was repeated a minimum of 3 times. The data (Fig. 7B) confirmed the inhibition caused by dopamine and epinine and showed that the effect was dose-dependent. In both cases, significant inhibition was observed at concentrations equal to or >10 µM.

The study was subsequently expanded to test other drugs that normally interact with BA receptors and were shown to have activity towards recombinant SmGPR-3 in the yeast assay. Substances were tested at 50 µM (flupenthixol, promethazine, epinine, dopamine) or 500 µM (adrenaline, metanephrine, haloperidol). With the exception of haloperidol, which had no significant motor effects, all the other substances produced significant changes (P<0.05) in motor activity compared to the untreated larvae (Fig. 7C). However the results do not show a correlation between the phenotypic effects of these drugs and those seen *in vitro* with the recombinant receptor. Adrenaline and its methylated derivative, metanephrine have weak agonist activity towards SmGPR-3 in the yeast assay, yet they were found to have opposite effects on larval motility. Adrenaline produced the same decrease in motility as dopamine and epinine, whereas metanephrine caused marked hyperactivation, increasing the frequency of body wall movements ≈3-fold. Among the antagonists of SmGPR-3, haloperidol had no effect but flupenthixol and promethazine both caused a significant decrease in motor activity. Drug-treated animals were routinely assayed for viability, using the methylene blue dye exclusion assay described by Gold [32]. There were no measurable effects on viability at the drug concentrations tested, though we cannot rule out some degree of drug-induced toxicity that would not be detected by this assay.

### SmGPR-3 homology modeling and ligand docking

**Figure 8:**
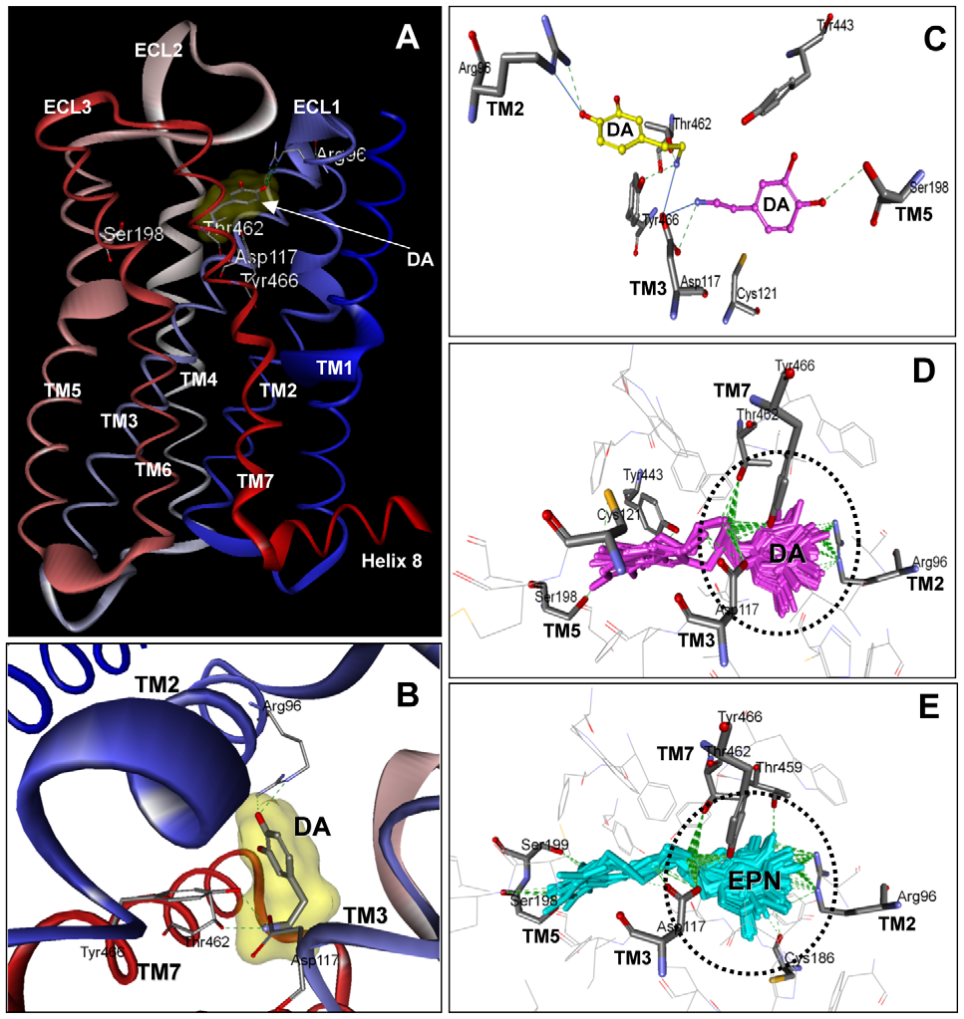
Homology modeling of SmGPR-3 and ligand docking. (A) A homology model of SmGPR-3 with bound dopamine (DA) is shown. The model was generated using the β-2 adrenergic receptor (PDB Accession # 2rh1) as a structural template, as described in the Methods. The positions of the 7 predicted transmembrane (TM) helices and 3 extracellular loops (ECL) are marked. The additional intracellular helix at the C-terminal end (helix 8) is also shown. Amino acid residues that are predicted to interact with dopamine include: Arg 96 (R^2.64^), Asp117 (D^3.32^), Thr462 (T^7.39^) and Tyr466 (Y^7.43^). (B) Close-up of the predicted binding pocket showing the best docking pose of dopamine (DA) and the principal ligand binding residues. (C) Two different docking poses of dopamine (DA) are shown. Note that the position of the catechol ring in the two conformations is reversed. In the best scoring pose (yellow), the ring hydroxyl interacts with Arg96 (R^2.64^) near the extracellular junction of TM2, whereas in the other docking pose (magenta) the ring interacts with Ser198 (S^5.42^) of TM5 instead. In both cases the protonated amino end of dopamine is anchored to Asp117 (D^3.32^) of TM3. An overlay of potential docking poses of dopamine (DA, panel D) and the structurally related ligand, epinine (EPN, panel E) shows the majority of interactions occurring with TM2, 3 and 7 for both ligands. The core interacting residues are shown and predicted H-bonds are marked by broken green lines.

Studies of mammalian BA receptors have localized the agonist binding pocket to a crevice formed by residues near the extracellular side of the TM helices, particularly TM3, 5, 6 and 7 [23], [36], [38]–[43]. Several ligand-interacting residues have been identified within this pocket. Of particular importance in catecholamine (dopamine, adrenergic) receptors is the aforementioned aspartate of TM3 (D^3.32^), which anchors the protonated amino group of the ligand, and three closely positioned serines in TM 5 (S^5.42^, S^5.43^, S^5.46^), which interact with the hydroxyl groups on the catechol ring. Three of these residues are also conserved in SmGPR-3 (D^3.32^, S^5.42^, S^5.43^) and therefore we questioned whether they might be involved in the interaction with dopamine. A homology model of SmGPR-3 was obtained from a structural alignment with the β2-adrenergic receptor (2rh1) and used for docking simulations. The hypothetical structure shows the typical topology of a Class A GPCR with 7 TM helices and one additional short helix (helix 8) at the C-terminal end that runs parallel to the membrane (Fig. 8A). Starting with this structure, we searched for potential binding cavities, using the site finder command in Discovery Studio. The analysis identified a possible site, which was located in the expected region of the receptor and included the canonical D^3.32^ of TM3 (residue Asp117). The dopamine structure was docked virtually onto this site and 240 docked ligand poses were examined and scored to search for the most favourable binding interactions. We identified approximately 20 residues located within 5 Å of the docked ligand, which are predicted to form the binding pocket of the receptor (Table S1). Among these residues there are five amino acids showing direct interactions with dopamine (Arg96/R^2.64^, Asp117/D^3.32^, Ser198/S^5.42^, Thr462/T^7.39^ and Tyr466/Y^7.43^) (Fig. 8A, B). The protonated amino group of dopamine is anchored to Asp117/D^3.32^ of TM3, as expected, and also forms additional H-bond interactions with Thr462/T^7.39^ and Tyr466/Y^7.43^of TM7. As for the catechol ring of dopamine, we observed more that one set of interactions depending on the orientation of the ligand. The most favourable orientation (highest docking scores) had the catechol ring interacting with Arg96/R^2.64^ of TM2 (Fig. 8C, yellow). Dopamine could also be docked in a different orientation where the catechol ring was pointed towards TM5 and interacted with one of the conserved serines of TM5, Ser198/S^5.42^ (Fig. 8C, magenta) but the docking scores were lower and we did not see interactions with the other conserved serine of TM5 (Ser199/S^5.43^). About 85% of the docked poses examined had interactions with Arg96/R^2.64^ of TM2 instead of Ser198/S^5.42^ (Fig. 8D). Since SmGPR-3 is as responsive to epinine as it is to dopamine, we repeated the docking simulation using epinine as the ligand. An overlay of all the potential docked poses shows that epinine binds to the same pocket as dopamine and interacts with the same core residues (Fig. 8E). Like dopamine, the most favourable epinine interactions involved residues of TM3 (Asp117/D^3.32^), TM2 (Arg96/R^2.64^) and TM7 (Thr462/T^7.39^ and Tyr466/Y^7.43^). We also observed less favourable interactions between the catechol ring of epinine and TM5, similar to dopamine, except in this case interactions occurred with both conserved serines, Ser198/S^5.42^ and Ser199/S^5.43^.

## Discussion

The *S. mansoni* genome encodes 16 GPCR-like sequences that share significant homology with aminergic receptors from other species [13]. A comparative bioinformatics analysis of these putative *S. mansoni* BA receptors with homologues of vertebrate and invertebrate origins enabled us to identify a new clade, the SmGPRs, which contains approximately half of all *S. mansoni* aminergic receptors and at least another two homologues of *S. japonicum* [15]. SmGPRs are easily recognized as Class A GPCRs due to their heptahelical organization, short N-terminus and the presence of signature motifs such as the DRY peptide (near TM3), FxxCWxxFF (TM6) and NPxxY (TM7) among others. Nevertheless, these receptors are considered novel; they are clearly more related to each other than they are to other BA receptors in the database, including other schistosome BA receptors. The dendogram analysis suggests that the SmGPRs diverged from a common ancestor early in evolution, probably through a series of gene duplications that gave rise first to the SmGPR-1/SmGPR-3 branch and subsequently the remaining sequences. We note that the SmGPR sequences grouped within this clade are all located in relatively close proximity to each other in the *S. mansoni* genome (Smp_scaff000103) [13], which is consistent with a mechanism of gene duplication. A distinctive feature of the SmGPRs is the absence of residue D^3.32^, which is replaced with an asparagine in all of these sequences except for SmGPR-3. As mentioned earlier, D^3.32^ is highly conserved in BA receptors [22], [36], [38], [39] and therefore the asparagine substitution marks a significant departure from current models of receptor structure. Thus far SmGPRs have been found only in *S. mansoni* and *S. japonicum*. We checked all the available planarian genomes as well as the general database at NCBI and found no other sequences that aligned within this clade. As more genomes become available, it will be of interest to determine if SmGPRs are present in other parasitic flatworms or if they are unique to schistosomes.

The functional expression analysis of SmGPR-3 was conducted in yeast cells. Yeast provides a robust expression system for recombinant, hard to express proteins. SmGPR-3 could not be expressed in mammalian cells (not shown) but it produced a functional receptor when expressed in the budding yeast *S. cerevisiae*. SmGPR-3 is the third member of this clade to be cloned and characterized. The first two (SmGPR-1 and -2) were found to be selectively activated by histamine when expressed *in vitro* [15], [25]. Given the structural similarity among these receptors, we questioned whether SmGPR-3 might also be responsive to histamine. However the activity assays revealed that SmGPR-3 was preferentially activated by catecholamines. The strongest response was obtained with dopamine and its methylated derivative, epinine, but the receptor also recognized noradrenaline and adrenaline, suggesting broad specificity for catechol derivatives. When tested against classical dopaminergic and other BA antagonists, SmGPR-3 exhibited a mixed pharmacological profile that did not conform to any known mammalian receptor. The most potent inhibitors of SmGPR-3 included two classical DA antagonists (haloperidol, flupenthixol), an anti-histaminic drug (promethazine) and a relatively nonselective inhibitor (cyproheptadine) that normally targets serotonin and histaminergic receptors. In contrast, anti-dopaminergics such as clozapine and spiperone either had little inhibitory effect or exhibited agonist activity. Interestingly, we noted that two of the most potent inhibitors of SmGPR-3, promethazine and flupenthixol, are also the most potent inhibitors of SmGPR-2 [15], suggesting that SmGPRs may have common pharmacological properties, even if the amine ligands are different.

To explore the role of SmGPR-3 in the bloodfluke, we produced a specific polyclonal antibody and then examined the tissue distribution of the receptor in different developmental stages of the parasite by confocal immunofluorescence. The results show that this receptor is predominantly neuronal and is expressed in the nervous system of both larvae (cercaria) and adult worms. The distribution of SmGPR-3 immunoreactivity in cercaria closely parallels that of catecholamine-containing neurons, which were previously described in *S. mansoni* and *S. japonicum* by biochemical methods [14]. In cercaria these neurons localize in part to the major longitudinal nerve cords and the posterior transverse commissure, sites that are also enriched in SmGPR-3. SmGPR-3 is probably activated by dopamine released from these centrally located neurons and mediates inter-neuronal dopamine signalling within the cercarial CNS. In the adults, SmGPR-3 is more widespread and localizes both to the CNS and elements of the PNS, suggesting a greater diversity of activities. As in the larvae, we detected strong immunoreactivity throughout the major longitudinal nerve cords and cerebral ganglia. In addition, the adult worms express high levels of SmGPR-3 outside the CNS, particularly in the smaller peripheral nerve fibers and plexuses that supply the body wall muscles and the suckers among other tissues. We do not know how this compares with the distribution of dopamine neurons in the adults, which has yet to determined. However there is evidence from other flatworms that dopamine neurons are present both in the CNS and the peripheral innervation of the somatic muscles, where dopamine is believed to play an important role in motor control [16], [18]. Our results thus suggest that SmGPR-3 has important activities both within and outside the CNS in adult worms. The prevalence of SmGPR-3 in the nerve fibers of the circular muscles strongly implicates this receptor in the control of muscle function and suggests this is one of the mechanisms by which dopamine controls movement of the worm.

The first evidence of dopaminergic activity in schistosomes dates back to the 1970s, when researchers reported that addition of dopamine caused a pronounced lengthening of the body in intact adult worms [44], [45]. Subsequent studies demonstrated that the lengthening effect was due to relaxation of the body wall muscles [21]. It was further suggested that dopamine had both direct and indirect effects on the musculature, possibly by acting through more than one receptor [21], though the molecular mechanisms were unknown. Our continuing investigation of this system is beginning to shed new light on these early observations. SmGPR-3 is the second dopamine receptor to be identified in *S. mansoni*. The tissue distribution of the other receptor, named SmD2 is quite different from that described here. SmD2 is absent in the CNS and it is enriched in the body wall muscles [22]. Although SmGPR-3 is also present in the body wall, it is associated with neuronal elements rather than the muscle per se; we did not see any co-labelling of SmGPR-3 with phalloidin, the counterstain that was used to probe the muscle layers. Thus, we conclude that there are at least two routes of dopaminergic motor control in *S. mansoni* involving both direct and indirect mechanisms, as was hypothesized in the earlier studies. One pathway is mediated by SmD2, which is predicted to act directly on the musculature, whereas the second is a more indirect neuronal pathway mediated by SmGPR-3. The localization of SmGPR-3 suggests that the receptor probably controls neuronal input to the body wall muscles, most likely by modulating release and/or signalling activity of another transmitter. This type of indirect activity is consistent with the notion that BAs can function both as neuromodulators and classical neurotransmitters. For example, in *C. elegans* dopamine controls motor activity indirectly through centrally located neurons that in turn modulate muscle function [46].

While it is well established that dopamine has inhibitory neuromuscular effects in schistosomes [21], the outcome of these effects on movement is unclear. Some early studies reported an increase in the length of the body but no effect on motility [44] whereas others reported both an increase in length and a decrease in motor activity when adult worms were treated with dopamine [45]. These studies were based on qualitative assessments of worm movement and did not include larval stages of the parasite. We have revisited this issue using a more quantitative imaging assay and *in vitro* transformed schistosomula, which are best suited for measurements of motility in culture. The results support the notion that dopamine has inhibitory motor effects in schistosomes; dopamine-treated larvae were significantly less motile than the controls. The inhibition was dose-dependent, starting at about 10 µM, and resulted in nearly complete paralysis at higher concentrations. Similar effects were observed in larvae treated with adrenaline and the dopamine metabolite, epinine, suggesting this is a common response to catecholamines. The inhibitory effect of dopamine described here is much stronger than that reported earlier for adult worms [44], [45] but, surprisingly, we did not see an overall increase in body length, which was a predominant response in the adults. This discrepancy could be due to differences in experimental protocol or it could be a function of the different developmental stages used in the studies, larvae versus adults. As mentioned earlier, the distribution of SmGPR-3 is more widespread in the adult worms than the larvae. SmGPR-3 localizes to the peripheral innervation of the somatic muscles in the adults whereas in the larvae it is more restricted to the CNS. It is possible the different dopamine-induced behaviours, paralysis versus elongation, are due to developmental changes in the expression patterns of these receptors.

Besides catecholamines, we repeated motility assays with a variety of substances that were shown to interact with the recombinant SmGPR-3 in the yeast system. Given that exogenous dopamine paralyzes the larvae, we had expected antagonists of this receptor to cause hyperactivity but that was not observed. One of the antagonists tested, haloperidol, caused a small increase in motility but this was not statistically significant, whereas the other two (flupenthixol and promethazine) had the opposite effect and caused marked inhibition of movement at the concentrations tested. A probable explanation for these results is that the drugs are interacting with more than one receptor in the intact parasite, for example the aforementioned SmD2 receptor, a SmGPR homologue, or others that have yet to be identified. The paralysis caused by promethazine and flupenthixol could be due to interactions with SmGPR-2, which is also strongly inhibited by these drugs [15]. Another consideration is the possibility of general (receptor-independent) toxicity effects. The drugs used in this study did not affect parasite viability but we cannot rule out other, more subtle effects that might have hindered motility and were not detected in this study. One of the more unexpected results of this survey was the effect of metanephrine on the larvae. Metanephrine is structurally related to adrenaline but it had the opposite effect on larval movement. Whereas adrenaline caused paralysis, the metabolite caused very strong hyperactivity, more than doubling the frequency of movement. Metanephrine was found to have weak agonist activity towards SmGPR-3 *in vitro* (not shown); it is possible that an excess of exogenous metanephrine is able to compete with endogenous dopamine (or adrenaline) for this receptor, thus decreasing endogenous signalling. Although the mode of action remains unclear, the results nonetheless show that catecholamine metabolites and other dopaminergic agents have strong effects on larval motility. The challenge for future studies is to determine which of the *S. mansoni* dopamine receptors, smGPR-3, SmD2 or others is targeted by these drugs and to identify more selective inhibitors that could be used to disrupt parasite movement.

The strong effects of epinine and metanephrine on larval motility raise interesting questions about the possible role of these substances in the parasite. In mammals, epinine is a naturally occurring but relatively minor by-product of DA metabolism. It is produced from DA by the activity of phenylethanolamine N-methyltransferase (PNMT) and can be further metabolized to adrenaline, though this reaction rarely occurs in mammals [47]. Metanephrine is one of the major metabolites of adrenaline in humans; it is synthesized through the action of catechol-O-methyl transferase (COMT) and it is normally excreted in the urine. Although they are considered to be inactive metabolites in higher organisms, epinine and metanephrine are biologically active in some protozoa and invertebrates. For instance, epinine replaces noradrenaline as the major substrate for adrenaline biosynthesis in the unicellular protozoan *Tetrahymena pyriformi* [48]. In the cnidarian, sea pansy *Renilla koellikeri*, epinine, metanephrine and another related metabolite, normetanephrine, are all present at high levels and are believed to be neuroactive [49]. It is unknown if these substances also occur in flatworms. We note, however, that at least some of the enzymes required for endogenous biosynthesis of these substances are present in schistosomes. The *S. mansoni* genome encodes a putative O-methyltransferase (CAZ32787, Smp_052470) that shares significant homology with COMTs from other species. If these metabolites are produced by the parasite, their interaction with SmGPR-3 and strong effects on larval motility could prove to be biologically important.

In the absence of a crystal structure, which is lacking for all but a few GPCRs, researchers often resort to homology models to explore the structural organization of new receptors. We have produced a homology model of SmGPR-3, using the human β2-adrenergic receptor as a structural template, and then performed virtual docking of dopamine onto the hypothetical structure. It has been published that dopamine/adrenergic receptors bind their BA ligands through the highly conserved D^3.32^ in TM3 and through one or more of three serines (S^5.42^, S^5.43^ and S^5.46^) present in TM5 [23], [36], [39]–[43]. SmGPR-3 has the conserved D^3.32^ of TM3 and two of the serines (S^5.42^ and S^5.43^) in TM5 but S^5.46^ is missing. We questioned whether a different residue might substitute for the missing serine or, given the novelty of the sequence, dopamine could interact with different residues entirely. Our docking simulation verified the interaction with D3.32 (Asp117) but suggests that the serines of TM5 are unlikely to play a major role in dopamine binding. Instead, we found potential binding sites in TM2 and TM7. One of the more interesting findings of this analysis is the predicted interaction with R^2.64^ (Arg96) of TM2. This arginine is not conserved in the mammalian dopamine receptors and the cognate residue (alanine or hydrophobic) is not known to be directly involved in dopamine binding. Another surprising difference is the apparent absence of ligand interactions with aromatic residues of TM6, which are conserved in SmGPR-3 (F^6.51^, F^6.52^) and would normally be expected to contribute to the binding of the catechol moiety. These differences must be viewed with caution, given the low sequence homology of SmGPR-3 compared to the template and the inevitable artefacts associated with homology models. Nevertheless, the results identify potentially important differences between the parasite and host receptors that can now be tested by mutagenesis and functional analyses. The predicted involvement of R^2.64^ is particularly noteworthy because of its location near the extracellular junction of TM2. The TM2 junction is a major component of the antagonist binding pocket in the human D3 receptor [40] and therefore this region could be important for the function and pharmacology of SmGPR-3. The fact that R^2.64^ is not conserved in the host (but is present in all the SmGPRs) further identifies this region as a potential target for the development of selective receptor blockers.

This study adds to a growing body of molecular evidence that points to dopamine as a major neurotransmitter of the schistosome nervous system. Besides SmGPR-3 and the previously described SmD2 receptor [22], researchers have characterized a dopamine biosynthetic enzyme in *S. mansoni* [50] and, more recently, discovered a high-affinity dopamine transporter [51], which is likely involved in the recycling/inactivation of the amine. The broad distribution of SmGPR-3 reported here suggests a great diversity of dopamine activities in this parasite, more than was previously believed. Although we have focussed our attention on neuromuscular and motor effects, it should be noted that SmGPR-3 was also found in the innervation of the caecum, the tubercles and, interestingly, the male reproductive system. Very little is known about the neuronal control of reproduction in schistosomes. We have previously identified a glutamate receptor in the female reproductive tract of *S. mansoni* [52] but, to our knowledge, this is the first evidence of a neurotransmitter receptor expressed in the male testes and it suggests a novel role for dopamine in this system. The presence of SmGPR-3 in the male tubercles is also noteworthy. Several neurotransmitters and neuronal proteins have been identified in the tubercles of schistosomes, where they are believed to be associated with sensory nerve endings [53]–[55]. SmGPR-3 expressed in these sites could play an important role in chemosensory signalling, either as part of an endogenous pathway or in response to exogenous (host-derived) catecholamines. More research is needed to clarify the nature of these effects and to elucidate the mode of action of dopamine in the parasites.

## Supporting Information

Figure S1 **Immunoprecipitation and western blot analysis of SmGPR-3.** SmGPR-3 was immunoprecipitated (IP) from a preparation of solubilized *S. mansoni* membranes, using a specific anti-SmGPR-3 peptide antibody that was covalently coupled to agarose beads. After washing, the bound proteins were eluted from the antibody beads under acidic conditions and immunoblotted (IB) with purified anti-SmGPR-3 antibody (duplicate samples), peptide-preadsorbed antibody or pre-immune serum. The sizes of relevant protein standards are indicated.
(TIF)

Figure S2 **Expression of SmGPR-3 in** ***S. mansoni*** **schistosomula.** (A) Quantitative PCR (qPCR) was performed on oligo-dT reverse-transcribed cDNA from *S. mansoni* schistosomula. The larvae were obtained from cercaria by mechanical transformation [27], [28] and were tested at 3 days and 8 days post-transformation with similar results. Real-time qPCR was performed as described previously [15], [52], using primers designed to amplify a SmGPR-3 product of 243 bp (forward: 5′-CCACTGGACAAATGATTGTATTG-3′; reverse: 5′-ACCATTCCATTGAAACATCCATTAC-3′) or a 206 bp fragment of *S. mansoni* glyceraldehyde-3-phosphate dehydrogenase (GAPDH, Accession # M92359), which was used as a housekeeping gene control (forward: 5′-GTTGATCTGACATGTAGGTTAG-3′; reverse: 5′-ACTAATTTCACGAAGTTGTTG-3′). The qPCR cycling conditions were as follows: 53°C/30 s, 94°C/2 min followed by 50 cycles of 94°C/15 s, 53°C/30 s and 72°C/30 s. The data show typical amplification curves for GAPDH and SmGPR-3 obtained from a single experiment in duplicates. Average (± SEM) Ct values were determined from 3 separate experiments, each in duplicate or triplicate: SmGPR-3, Ct = 23.2±2.3; GAPDH, Ct = 19.5±2.0. (B) Conventional end-point RT-PCR was performed with RNA obtained from 3 day-old schistosomula, using PCR primers designed to amplify a 461 bp fragment of SmGPR-3 (positions 1–461). A band of the correct size was observed in samples containing oligo-dT reverse-transcribed cDNA (lane 1) but not a negative control that lacked reverse transcriptase (lane 3). As a positive control, the PCR was repeated with the cloned SmGPR-3 cDNA (pGEM-T/SmGPR-3 plasmid) as a template and the same size band was observed (lane 2).
(TIF)

Table S1 **Binding site residues interacting within 5 Å of the ligand.**
(DOC)
