# Supplementary figures and images for: A Novel G Protein-Coupled Receptor of Schistosoma mansoni (SmGPR-3) Is Activated by Dopamine and Is Widely Expressed in the Nervous System
Source: PLoS Negl Trop Dis. 2012 Feb 28;6(2):e1523. doi: 10.1371/journal.pntd.0001523 (PMC3289605; doi:10.1371/journal.pntd.0001523)

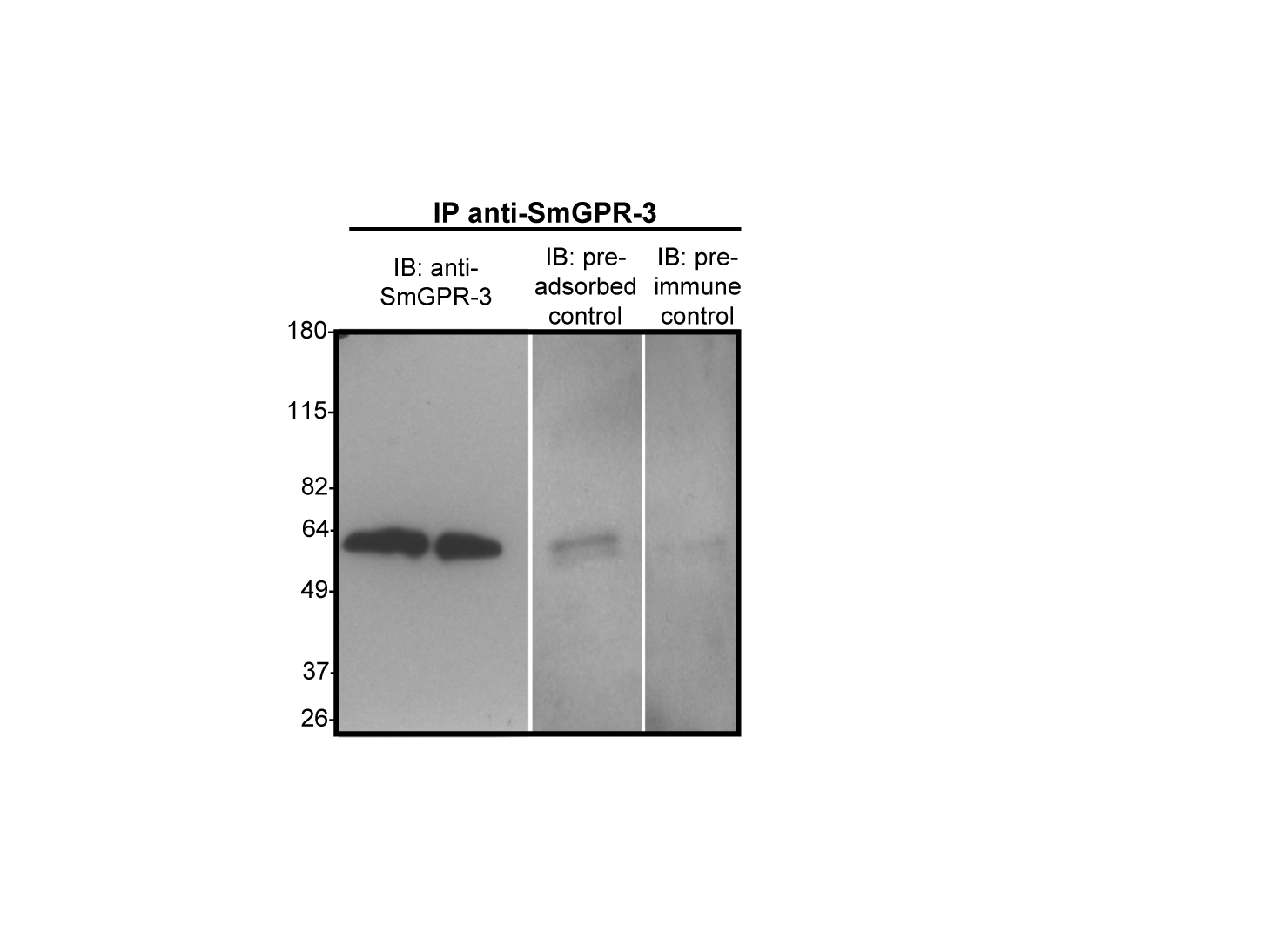

Supplement: Figure S1 — Immunoprecipitation and western blot analysis of SmGPR-3. SmGPR-3 was immunoprecipitated (IP) from a preparation of solubilized S. mansoni membranes, using a specific anti-SmGPR-3 peptide antibody that was covalently coupled to agarose beads. After washing, the bound proteins were eluted from the antibody beads under acidic conditions and immunoblotted (IB) with purified anti-SmGPR-3 antibody (duplicate samples), peptide-preadsorbed antibody or pre-immune serum. The sizes of relevant protein standards are indicated. (TIF) [file pntd.0001523.s001.tif]

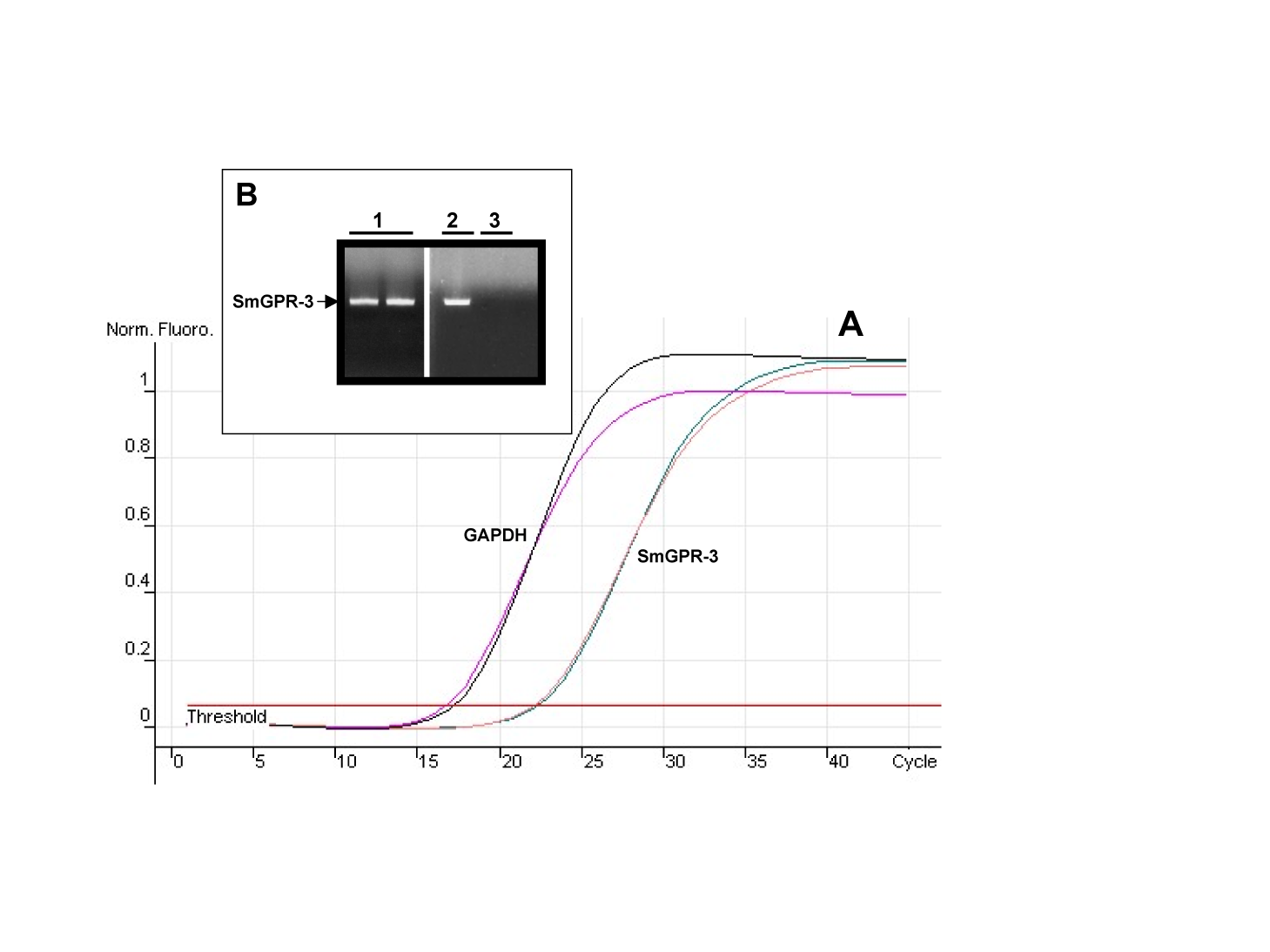

Supplement: Figure S2 — Expression of SmGPR-3 in S. mansoni schistosomula. (A) Quantitative PCR (qPCR) was performed on oligo-dT reverse-transcribed cDNA from S. mansoni schistosomula. The larvae were obtained from cercaria by mechanical transformation [27], [28] and were tested at 3 days and 8 days post-transformation with similar results. Real-time qPCR was performed as described previously [15], [52], using primers designed to amplify a SmGPR-3 product of 243 bp (forward: 5′-CCACTGGACAAATGATTGTATTG-3′; reverse: 5′-ACCATTCCATTGAAACATCCATTAC-3′) or a 206 bp fragment of S. mansoni glyceraldehyde-3-phosphate dehydrogenase (GAPDH, Accession # M92359), which was used as a housekeeping gene control (forward: 5′-GTTGATCTGACATGTAGGTTAG-3′; reverse: 5′-ACTAATTTCACGAAGTTGTTG-3′). The qPCR cycling conditions were as follows: 53°C/30 s, 94°C/2 min followed by 50 cycles of 94°C/15 s, 53°C/30 s and 72°C/30 s. The data show typical amplification curves for GAPDH and SmGPR-3 obtained from a single experiment in duplicates. Average (± SEM) Ct values were determined from 3 separate experiments, each in duplicate or triplicate: SmGPR-3, Ct = 23.2±2.3; GAPDH, Ct = 19.5±2.0. (B) Conventional end-point RT-PCR was performed with RNA obtained from 3 day-old schistosomula, using PCR primers designed to amplify a 461 bp fragment of SmGPR-3 (positions 1–461). A band of the correct size was observed in samples containing oligo-dT reverse-transcribed cDNA (lane 1) but not a negative control that lacked reverse transcriptase (lane 3). As a positive control, the PCR was repeated with the cloned SmGPR-3 cDNA (pGEM-T/SmGPR-3 plasmid) as a template and the same size band was observed (lane 2). (TIF) [file pntd.0001523.s002.tif]
