# Supplementary material for: A Novel G Protein-Coupled Receptor of Schistosoma mansoni (SmGPR-3) Is Activated by Dopamine and Is Widely Expressed in the Nervous System
Source: PLoS Negl Trop Dis. 2012 Feb 28;6(2):e1523. doi: 10.1371/journal.pntd.0001523 (PMC3289605; doi:10.1371/journal.pntd.0001523)
Supplement: Table S1 — Binding site residues interacting within 5 Å of the ligand. (DOC) [file pntd.0001523.s003.doc]

Table S1: Binding site residues interacting within 5 Å of the ligand

| **Domain** | **Residue** |
| --- | --- |
| TM 2 | Val89, Ala93, **Arg96*** |
| ECL1 | Trp102 |
| TM 3 | His113, Ile114, **Asp117*,** Cys121 |
| ECL 2 | Tyr187, Ile188 |
| TM5 | **Ser198***, Ser199, Ala202 |
| TM 6 | Trp436, Phe439, Phe440 |
| TM 7 | Tyr458, **Thr459***,**Thr462***, Trp463, **Tyr466*** |

Dopamine was docked onto the SmGPR-3 homology model, as described in the Methods. The residues shown above are predicted to contribute to the dopamine binding pocket of SmGPR-3. Residues that form direct interactions with the ligand are marked with an asterisk in bold. TM, transmembrane domain; ECL, extracellular loop.
